# Slice-selective implementation of an adiabatic T_2_Prep sequence increases coronary artery conspicuity at 3T

**DOI:** 10.1186/1532-429X-14-S1-O52

**Published:** 2012-02-01

**Authors:** Sahar Soleimanifard, Michael Schär, Jerry L Prince, Robert G Weiss, Matthias Stuber

**Affiliations:** 1 Johns Hopkins University, Baltimore, MD, USA; 2 Philips Healthcare, Cleveland, OH, USA; 3 University of Lausanne, Lausanne, Switzerland

## Background

In non-contrast coronary MRA, the non-selective T_2_Prep^1-2^ has been widely used for contrast enhancement between the coronary blood-pool and the myocardium^3-5^. This non-selective pre-pulse affects the magnetization both inside and outside the field-of-view, resulting in a reduced steady-state magnetization of the inflowing blood, and consequently a penalty in SNR. We hypothesize that a slice-selective T_2_Prep would leave the magnetization of blood outside the imaged volume unaffected, and thereby minimize SNR penalty for inflowing blood. The purpose of this work was to implement a slice-selective T_2_Prep coronary MRA sequence and to assess the SNR gain quantitatively.

## Methods

### Simulations

bloch equations were simulated to test the above hypothesis and to estimate the expected SNR gain.

### Implementation

the selective T_2_Prep was implemented by replacing the non-selective 90° RF pulses with slice-selective versions plus flow-compensating gradients, and an additional time gap between the T_2_Prep and other pre-pulses to allow for inflow of a larger volume of spins with equilibrium magnetization (Figure1). The T_2_Prep volume was graphically selected along the arterial axis and orthogonal to the imaged volume without covering the ventricles (Figure 1c) to avoid saturation of the inflow spins in the ascending aorta.

### Experiments

volume-targeted 3D free-breathing navigator-gated and corrected coronary MRA^5^ were acquired on a Philips Achieva 3T a) without T_2_Prep, b) with the conventional T_2_Prep, and c) with the proposed slice-selective T_2_Prep (Tgap=150ms) in 10 healthy volunteers. Repeated measures of blood SNR, vessel-neighborhood CNR, and vessel sharpness were statistically compared using ANOVA with Tukey post hoc test.

## Results

Consistent with the hypothesis, numerical simulations suggest that the slice-selective T_2_Prep leads to an SNR increase in the blood-pool (Figure 1A) when compared to its non-selective counterpart. Figure 1C shows an *in vivo* result obtained with the slice-selective T_2_Prep localized along the dark bar (arrows). When compared to the non-selective T_2_Prep, vessel sharpness improved significantly using the proposed implementation (54.1±2.4% vs. 48.5±2.4%, p<0.05, Figure 2A). While both T_2_Prep variants led to an SNR penalty when compared to no T_2_Prep as previously reported^3^, the slice-selective T_2_Prep still led to a significantly improved SNR compared to conventional T_2_Prep (39.9±3.2 vs. 27.7±2.5, p<0.02, Figure 2B). A similar finding was observed in CNR measurements (16.1±1.6 vs. 11.8±1.5, p<0.05, Figure 2C).

## Conclusions

A slice-selective adiabatic T_2_Prep leads to improved coronary artery conspicuity when compared to its conventional non-selective counterpart.

## Funding

This work was supported in part by the NIH/NHLBI research grants.

RO1HL084186, ARRA 3R01Hl084186-04S1.
